# Understanding grapevine-microbiome interactions: implications for viticulture industry

**DOI:** 10.15698/mic2015.05.204

**Published:** 2015-05-04

**Authors:** Iratxe Zarraonaindia, Jack A. Gilbert

**Affiliations:** 1 Argonne National Laboratory, Institute for Genomic and Systems Biology, Argonne, Illinois, USA.; 2 Department of Genetics, Physical Anthropology and Animal Physiology, University of the Basque Country (UPV/EHU), Leioa (Bizkaia), Spain.; 3 Department of Ecology and Evolution, University of Chicago, Chicago, Illinois, USA.; 4 Department of Surgery, University of Chicago, Chicago, Illinois, USA.; 5 Marine Biological Laboratory, Woods Hole, Massachusetts, USA.; 6 College of Environmental and Resource Sciences, Zhejiang University, Hangzhou, China.

**Keywords:** Merlot microbiome holobiont, biotechnological applications, Terroir

## Abstract

Until recently, the analysis of complex communities such as that of the grapevine-microbe holobiont has been limited by the fact that most microbes are not culturable under laboratory conditions (less than 1%). However, metagenomics, the study of the genetic material recovered directly from environmental samples without the need for enrichment or of culturing, has led to open an unprecedented era in the field of microbiology. Importantly, this technological advance has now become so pervasive that it is being regularly applied to explore soils and plants of agricultural interest. Interestingly, many large companies are taking notice, with significant financial investment being used to exploring ways to manipulate the productivity, disease resistance and stress tolerance for crops by influencing the microbiome. To understand which microbes one needs to manipulate to influence this valuable characteristics, we need to sequence the microbiome and capture the genetic and hence functional metabolic information contained therein. For viticulture and other agricultural fields where the crop is also associated to particular flavor properties that may also be manipulated, understanding how the bacteria, fungi and viruses influence the development and hence chemical makeup of the crop is essential.

 Microbes associated with the plant meanwhile it is growing and producing may impact the organoleptic properties of products such as wine and are of major interest to viticulturists. Uncovering new ways to manage these properties as well as the crop yield may have a significant influence on the industry as well as for any given grower. Importantly, in a world scared of genetic modification, manipulating the microbial component of plants offers a non-GM way to enhance crop properties. Knowledge of the biogeography patterns and spatio-temporal dynamics of the grapevine associated microorganisms is fundamental if viticulturist want to be able to control/predict “bad years" and manage their crops effectively with this new tool. The ability to alter these properties hold the promise of creating a wine with viticulturists desired properties that might eventually be able to reproduce regional* Terroir* in any area; e.g. creating a Rioja out of Spanish vineyards.

Although grape-associated taxa are potentially important and likely have a direct effect on wine, the factors that shape the community in other plant structures and the soils grapevines are planted in, may also be of relevance. These taxa are likely to play a significant role in changing the plants accessibility to nutrients such as phosphate and nitrate. They also play a role in pathogen defense and alter the hormonal activity of the plant thereby affecting its stress tolerance. However, the grape has dominated our research for a long time and virtually nothing is known about the microbial dynamics at other plant organs. Our recent study created a baseline survey of the microbial structure and functional potential of the microbiota associated with grapevine organs and soil in order to understand the connectivity and colonization patterns among different plant niches. The long-term aim of these investigations is to determine whether these communities affect the wine properties that are sought after, and can be manipulated to control these characteristics.

We employed amplicon and shotgun metagenomic sequencing to study the taxonomy and functional potential of bacteria communities of merlot organs, both above- and below-ground. These included the grapes, leaves, flowers and roots. But we also explored the microbiome of the surrounding soil, both soils that were somewhat distant from the plant and the soil located in the immediate root zone. Using these techniques we interrogated small-scale biogeographic and temporal changes of the grapevine and vineyard associated microbiome by collecting samples from 5 vineyards from Long Island, NY (USA) over two years. We also explored how the microbial community changed in association with the cultivar of the grapevine, the physical and chemical characteristics of the soil, and the developmental stage (whether the vine was dormant, flowering, or ripe with fruit).

Our study demonstrated that each vine organ harbors an identifiable microbial community and functional capability presumably derived from the physicochemical environment and organ physiology constraints, as well as due to microbial competition. The biggest difference, as might be expected, was between the above and below ground organs, which were highly differentiated by both the bacterial composition and their potential function. Each of the niches maintained microbes associated with plant health, stress protection, productivity and plant development. For instance, *Sphingomonas* and *Pseudomonas*, known to show adaptation to aboveground nutrient- and water-limited ecosystems, were abundant in leaves and grapes. These two genera are also associated with disease suppression (*Sphingomonas*) and with water stress protection (*Pseudomonas*) in plants. In addition, members of the Rhizobiales order were abundant in belowground niches, where it is possible they play a role in N fixation, plant growth promotion and antibiotic production. The genes, and associated metabolic pathways, of these bacteria are likely to be of great interest to companies looking to develop plant probiotics for viticulture to improve crop performance, nutritional uptake efficiency and disease resistance. We demonstrated that grapes and leaves maintained bacteria that were predicted to have a wide range of xenobiotic degradation pathways, and targeting these different bacteria, or even just their genes, may yield significant biotechnological potential for attenuating environmental stressors such as the application of pesticides and herbicides.

Despite these potentially useful outputs, there is, as usual, a word of caution. Shotgun metagenomics is becoming extremely popular, however soil and root compartments are highly diverse, and so, ones need to sequence very deeply to be able to reconstruct the genes and pathways of interest which takes a lot of expertise and high investment of money and time. The organisms associated with the above ground tissues might be expected to be easier to characterize and therefore manipulate, as they are more selective environments, and hence are much less diverse. However, despite this low diversity, metagenomic sequencing of these tissues yields a massive overrepresentation of the host genomic material, which represents a technical limitation. Ideally you would like to only sequence the genetic material of the bacteria, so it becomes necessary to engineer ways to isolate the bacteria away from host tissues and then sequence them, however this biases the interpretation of that data by altering the structure of the microbial community. This is an area ripe for development.

Despite niche microbiome differences, there is connectivity among different compartments, where microbes colonize different organs either from within or from external to the plant. Endophytes are found living within the tissues, between the cells of the plant, whereas epiphytes are found living on the external surfaces of the plant. Environmentally driven changes in the epiphyte microbiome can influence the endophyte microbiome structure. Our results suggested that for all but a few bacteria, the soil was the primary reservoir. Bacteria from the soil colonized the plant probably by the roots, or by dust deposition, and those that came in via the roots move through the plant to colonize every tissue. In this regard, Merlot grapevine roots recruit several particular taxa with specific functions of benefit for the plant, whereby traits identified as favoring the movement or attraction of bacteria towards plant exudates and genes encoding the metabolism of macro- and micronutrients as well as plant stress tolerance were significantly more abundant in the root microbiome compared with the surrounding soil. A better understanding of the genes responsible for plant endophytic colonization could provide a knowledge base for biotechnology, and potentially form the basis for engineering more efficient plant-bacteria interactions. Soil microbes were also the main inoculum for the epiphytic microbiome. For instance, in this study Suffolk region merlot microbial communities were compared to those of Californian Merlot must and we found that the must samples shared more species with the soil environment than with grapes (despite the different geographic area of collection). This suggests that a lot of bacteria from the soil find themselves in the grapes, or that dust clings to the grape during harvesting. Either way, this implies that the soil microbiota is present during the first stages of grape fermentation, and may therefore play a more direct role in shaping the flavor characteristics of the wine product.

The debate of whether there is a microbiological component in the *Terroir* (the special characteristics that the land and climate imparts to the wine that makes it unique to a specific region) is mute. While viticulturists presumed that the *Terroir* comes from the land characteristics, our data just highlighted the importance of the soil microbiome (rather than soil physicochemical properties) in shaping the microbiology of the vine, and possibly directly shaping the characteristics of the wine. This is of high economic relevance, as it will open the possibility of reproducing a wine *Terroir* at any location a priori not suitable to generate that specific wine; potentially this could be achieved simply by inoculating certain soil microbes onto harvested grapes.

